# Disentangling host-parasite-pathogen interactions in a varroa-resistant honeybee population reveals virus tolerance as an independent, naturally adapted survival mechanism

**DOI:** 10.1038/s41598-019-42741-6

**Published:** 2019-04-17

**Authors:** Srinivas Thaduri, Jörg G. Stephan, Joachim R. de Miranda, Barbara Locke

**Affiliations:** Department of Ecology, Swedish University of Agricultural Sciences, 75007 Uppsala, Sweden

**Keywords:** Evolutionary ecology, Virology

## Abstract

The ectoparasitic mite, *Varroa destructor*, is unarguably the leading cause of honeybee (*Apis mellifera*) mortality worldwide through its role as a vector for lethal viruses, in particular, strains of the *Deformed wing virus* (DWV) and *Acute bee paralysis virus* (ABPV) complexes. This multi-level system of host-parasite-pathogen interactions makes it difficult to investigate effects of either the mite or the virus on natural host survival. The aim of this study was to remove confounding effects of varroa to examine the role of virus susceptibility in the enhanced survival of a naturally adapted Swedish mite-resistant (MR) honeybee population, relative to mite-susceptible (MS) honeybees. Caged adult bees and laboratory reared larvae, from varroa-free colonies, were inoculated with DWV and ABPV in a series of feeding infection experiments, while control groups received virus-free food. Virus infections were monitored using RT-qPCR assays in individuals sampled over a time course. In both adults and larvae the DWV and ABPV infection dynamics were nearly identical between MR and MS groups, but MS adults suffered significantly higher mortality than MR adults. Results suggest virus tolerance, rather than reduced susceptibility or virus resistance, is an important component of the natural survival of this honeybee population.

## Introduction

Despite the current popular and scientific focus on the possible role of agrochemical exposure on wild and managed bee health^1,2^, the ectoparasitic mite, *Varroa destructor*, together with its associated viruses, remains unarguably the leading cause of honeybee (*Apis mellifera*) colony mortality world-wide^3–5^. Two virus-complexes in particular, the *Deformed wing virus* (DWV) complex (including major strains DWV-A, DWV-B and DWV-C^6^) and the *Acute bee paralysis virus* (ABPV) complex (including major strains ABPV, *Kashmir bee virus* (KBV) and *Israeli acute paralysis virus* (IAPV)^7^), are transmitted highly efficiently by varroa mites, with devastating, though different, consequences^8^. Of the two virus complexes, DWV has become the most common and wide spread virus^9^ due to its superior adaptation to the combined selection pressures of varroa-mediated transmission, beekeeper mite management and colony winter survival^8^. Both DWV and ABPV are single-stranded RNA viruses that infect all stages of honeybee development^7,10^. In the absence of varroa mites, they are maintained in the colony at low levels as largely innocuous infections through a variety of horizontal and vertical transmission routes^11–16^. The characteristic symptoms of DWV are severe wing deformities resulting in flightless adults that die shortly after emerging^10^. However, these symptoms are almost exclusively associated with varroa-mediated transmission of DWV when the mite feeds on the bee during the pupal developmental stages^10,17^. ABPV, when transmitted by varroa mites, is characterized mostly by severe pupal mortality, and at elevated titres in adults by trembling, paralysis and behavioural inadequacies^7^.

The mite population in an infested honeybee colony can grow at an exponential rate, rapidly leading to an ABPV and/or DWV epidemic that ultimately results in the death of the colony^8,14^. For this reason, beekeepers need active mite control strategies to keep honeybee colonies alive in almost all parts of the world where the mite exists^3^. However, different honeybee populations naturally have varying responses to the pressure of this parasite. While most honeybees are susceptible to varroa mite parasitism and will typically die within a few years of an uncontrolled mite infestation^3^, some populations have been documented to survive without mite control measures and without the harmful effects typically associated with varroa mite infestation^18^. For example, mite control is not required in Africa or in parts of South America where the local *A*. *mellifera* populations or sub-species are effectively mite-resistant and maintain lower mite infestation levels than their relatives in other parts of the world^18,19^. There are also a few unique populations of honeybees in Europe and North America that have survived for extended periods without mite control^18,20^. One of the most well studied varroa mite surviving honeybee populations is on the island of Gotland, Sweden^21–24^. Through a natural selection process this population has adapted mite-resistant traits that reduce the mite’s reproductive success^22,24^.

Honeybee populations can likewise have varying responses to the virus infections vectored by the mite. A recent study on the Gotland mite-resistant population documented their survival with high levels of DWV infections while local mite-susceptible honeybee colonies, with similarly high DWV infections, all died^23^. Additionally, this population also appears to be resistant, at the colony level, to other viruses that are not necessarily directly transmitted by varroa mites, but that nevertheless harm honeybee health and reduce long-term survival^23^.

Resistance is the ability of the host to reduce the parasitic/pathogenic burden, whereas tolerance is defined as the ability of the host to reduce the harm done by the parasite/pathogen (in this case the disease-induced mortality)^25^. Honeybees, the varroa mite, and honeybee-infecting viruses that are also vectored by the mite, form a complex multi-level system of host-parasite-pathogen interactions, making it difficult to separate specific effects on the host when all three occur together. In order to better understand natural host survival mechanisms to uncontrolled mite infestation it is important to differentiate the extent to which they are due to resistance/tolerance to the mite, the viruses transmitted by the mite, or both.

The aim of this study was to assess the possible contribution of virus resistance/tolerance to the enhanced survival of the Gotland mite-resistant (MR) honeybee population independent of the confounding effects of varroa mite parasitism. We removed the direct influence of varroa on virus titres and compared the susceptibility of individual larvae and newly emerged adult bees from the Gotland mite-resistant (MR) population to oral ABPV or DWV infections, relative to that of bees from a mite-susceptible (MS) population, in laboratory virus infection time-course studies. Virus susceptibility was determined by comparing the virus titres of virus-inoculated bees relative to both the pre-experiment background virus titres and the natural infection development in uninoculated control bees, across the time-course. This formed the basis for comparing the virus susceptibilities of the MR and MS populations, which were each represented by bees from multiple, independent colonies. For the adult infection experiment, the mortality of the bees over time was also recorded, as well as the virus titres in dead bees.

## Results

### Larval infection experiment

**Figure 1:**
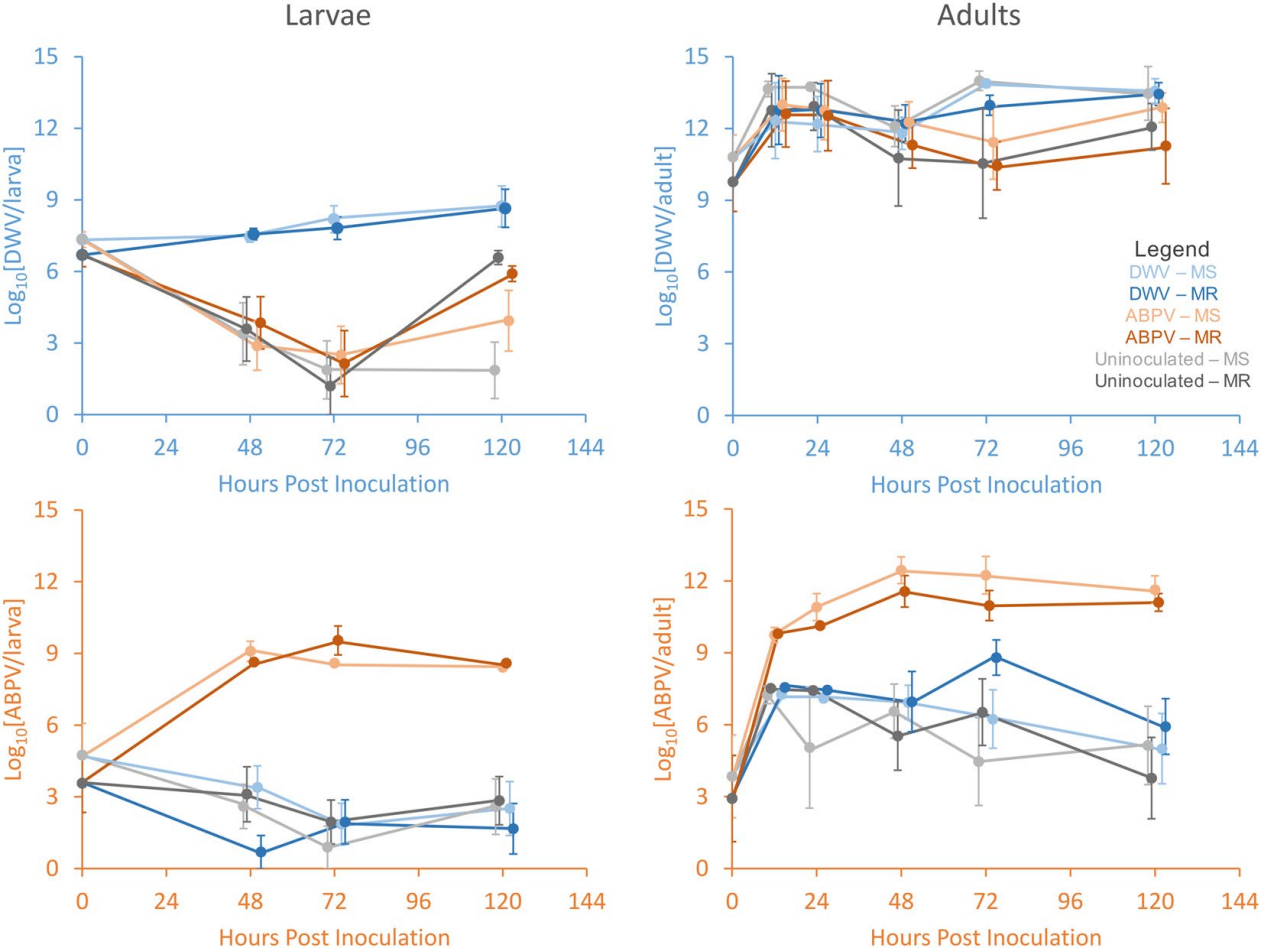
Graphical representation of the raw data from the deformed wing virus (DWV; blue panels - top) and acute bee paralysis virus (ABPV; orange panels - bottom) inoculation experiments in honeybee larvae (left panels) and adult worker bees (right panels). The line diagrams represent bees inoculated with either DWV (blue lines), ABPV (orange lines) or uninoculated bees (grey lines) from either mite resistant (MR; dark-shaded colours) or mite-susceptible (MS; light-shaded colours) colonies. Shown are the average DWV (blue) and ABPV (orange) virus titres plus standard errors across all replicate trials and colonies of each population, in relation to the time post-inoculation (hours).

No differences were detected between the MR and MS larvae in either DWV or ABPV susceptibility at any time during the experimental time course (Fig. 1; Table 1; Fig. S1). Additionally, no significant differences were detected between the MR and MS larvae in DWV or ABPV background infection levels, at 0 hpi, before the experiment started (Fig. 1; Table 2). Both the DWV and ABPV inoculations were successful at establishing an infection, in that the virus titres increased after the zero time point and was significantly elevated throughout the time-course relative to the uninoculated controls (Fig. 1; Table 1). For the DWV infection experiment, there was a slight increase in titres with time after 24 hpi (Fig. 1), suggesting the infection had not yet reached a plateau phase. For the ABPV infection experiment, there was no further increase in ABPV titres after 24 hpi (Fig. 1), suggesting that the infection reached a maximum upper limit at this time point.

**Table 1:** DWV and ABPV titres compared to uninoculated individuals. Analysis-of-deviance tables (Type III test) from linear mixed models investigating the effect of the treatment (inoculated/uninoculated, with the respective virus) and the colony history (MR/MS) on the virus titres over time for larvae at 24, 72 and 120 hours post inoculation (HPI) and for adults at 12, 24, 48, 72 and 120 HPI. Non-significant terms (italicized) were removed stepwise until the final minimal adequate model starting from the bottom row. Replicates nested within colony ID was included in all models as a random effect. Required transformations are indicated for each response variable.

Model ID	Life stage	Response Variable	Explanatory Variables	Χ^2^	Df	AICc	con.R^2^	p-value
1	Larvae	DWV^0.1^	HPI	5.67	2	385.30	0.48	0.06
			Treatment	67.18	1			<0.001
			*Colony history*	*0*.*39*	1	*386*.*68*	*0*.*48*	*0*.*53*
			*HPI × Treatment*	*4*.*30*	2	*382*.*93*	*0*.*50*	*0*.1*1*
			*Colony history × Treatment*	*1*.*17*	*1*	*38*2.*39*	*0*.*51*	*0*.2*7*
			*Colony history × HPI*	*2*.1*9*	2	*38*1.2*3*	*0*.*52*	*0*.*33*
			*Colony history × HPI × Treatment*	1.*66*	*2*	*378*.1*6*	*0*.*52*	*0*.4*3*
2	Larvae	ABPV^0.075^	HPI	1.95	2	252.47	0.76	0.37
			Treatment	74.01	1			<0.001
			HPI × Treatment	6.86	2			0.03
			*Colony history*	*0*.*82*	1	*255*.*03*	*0*.*77*	*0*.*36*
			*Colony history × HPI*	*1*.*79*	*2*	*257*.*59*	*0*.*77*	*0*.4*0*
			*Colony history × Treatment*	*0*.*00*	*1*	*260*.*15*	*0*.*76*	*0*.*96*
			*Colony history × HPI × Treatment*	*1*.*61*	*2*	*260*.*59*	*0*.*76*	*0*.44
3	Adults	DWV^0.3^	HPI	16.22	4	2208.81	0.60	<0.01
			*Colony history*	*0*.*761*	*1*	*2188*.4*7*	*0*.*63*	*0*.*38*
			*Treatment*	*0*.*2*4	1	2*171*.*5*2	*0*.*63*	*0*.*6*2
			*Colony history × HPI*	*6*.*77*	*4*	*2090*.*23*	*0*.*66*	*0*.*14*
			*Colony history × Treatment*	*1*.*09*	*1*	*2071*.*39*	*0*.*66*	*0*.*29*
			*HPI × Treatment*	*1*.*38*	*4*	*1996*.*89*	*0*.*65*	*0*.*84*
			*Colony history × HPI × Treatment*	*1*.*65*	*4*	*1917*.*74*	*0*.*65*	*0*.*79*
4	Adults	ABPV^0.075^	HPI	19.54	4	394.44	0.63	<0.001
			Treatment	3.65	1			0.05
			Colony history	0.13	1			0.71
			HPI × Treatment	11.164	4			0.02
			*Colony history × Treatment*	*0*.*002*	*4*	*396*.*19*	*0*.*62*	*0*.*95*
			*Colony history × HPI*	*0*.*09*	*4*	*400*.*81*	*0*.*61*	*0*.*99*
			*Colony history × HPI × Treatment*	*4*.*15*	*4*	*397*.*15*	*0*.*61*	*0*.*38*

**Table 2:** Analysis-of-deviance tables (Type III test) from linear mixed models that investigate the effect of colonies history (MR, MS) on the DWV and ABPV titers before the infection. Required transformations of the response variable are specified.

Model ID	Life stage	Response Variable	Explanatory Variables	Χ^2^	Df	AICc	con.R^2^	p-value
5	Larvae	DWV	Colony history	1.37	1	332.10	0.98	0.24
6		ABPV^0.125^	Colony history	0.59	1	59.44	0.06	0.44
7	Adults	DWV^−0.025^	Colony history	0.67	1	−6.08	0.06	0.41
8		ABPV^0.375^	Colony history	0.76	1	165.89	0.07	0.38

Furthermore, no significant difference in DWV titres was detected between the uninoculated control larvae and the ABPV-inoculated larvae (Fig. 1; Table S1). *Vice versa*, there was no significant difference in ABPV titres between the uninoculated control larvae and the DWV-inoculated larvae (Fig. 1; Table S1). This suggests that the two viruses do not affect each other’s infection dynamics, and effectively behave independently.

The final observation is that for both DWV and ABPV, the titres in the uninoculated controls (and in the larvae inoculated with the alternate virus) decrease over time from the zero time-point, with a low after 72 hpi, after which they increase again, more markedly for DWV, at 120 hpi (Fig. 1). It is as much this decrease in background infection levels over time as it is the inoculation/infection that is responsible for the significant difference throughout the time-course between the inoculated larvae and the uninoculated/alternate virus-inoculated controls (Fig. 1; Table 1; Fig. S1).

### Adult infection experiment

Whereas in the larval infection experiments the background titres of both DWV and ABPV in the uninoculated/alternate virus-inoculated controls tended to decrease with time (with a late upswing), in the adult infection experiments the reverse is true, in that the background DWV and ABPV titres in the controls tended to increase during the early part of the time-course, with a slight subsequent decrease (Fig. 1). The background DWV titres in newly hatched adult bees prior to inoculation, at 0 hpi, was moreover rather high, in both the MR bees and MS bees, making it difficult to determine whether DWV inoculation had any additional effect on the DWV titres (Fig. 1; Table 1; Table 2). It is only from 72 hpi onwards that the DWV-inoculated bees start diverging upwards from the uninoculated/ABPV-inoculated controls and show marginal evidence that the DWV-inoculation has, in fact, established an infection (Fig. 1). The ABPV inoculation experiment shows much earlier and much clearer separation in ABPV titres between the ABPV-inoculated bees and the uninoculated/DWV-inoculated control bees, helped in no small part by the very low initial background ABPV titres in newly hatched bees, at 0 hpi, demonstrating an infection developed due to inoculation (Fig. 1; Table 1; Table 2).

**Figure 2:**
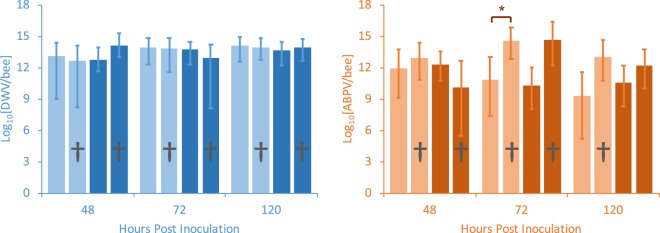
Titres of DWV (blue panel) and ABPV (orange panel) found at different time intervals post-inoculation in live and dead (†) adult honeybee worker bees from either MS (light-shaded colours) or MR (dark-shaded colours) bee colonies. Shown are predicted marginal means with 95% confidence limits from the respective models that still included all explanatory variables and their interactions. Statistically significant differences between live and dead bees are indicated by an asterisk.

As with the larval infection experiments, there was no significant difference between MR and MS bees in any of these time-course patterns, for either the DWV infection experiment or the ABPV infection experiment (Fig. 1; Table 1; Fig. S1), or for both the inoculated bees and the uninoculated/alternate virus-inoculated control bees (Fig. 1; Table 1; Table S1). Titres of DWV and ABPV did not differ between the colony histories before the experiments (Table 2). Also similar to the larval infection experiments, there was no major difference in the DWV time-course between the uninoculated and ABPV inoculated, nor in the ABPV time-course between uninoculated and DWV-inoculated bees, suggesting again that the two viruses have little effect on each other’s infection dynamics (Fig. 1; Table S1; Fig. S2). No differences were detected in DWV titres between the dead and the live adult bees (Fig. 2; Table 2). A similar result was observed with ABPV except that at the 72 hpi sampling point ABPV titres were slightly higher in dead adults than in live adults (Fig. 2; Table 2).

**Figure 3:**
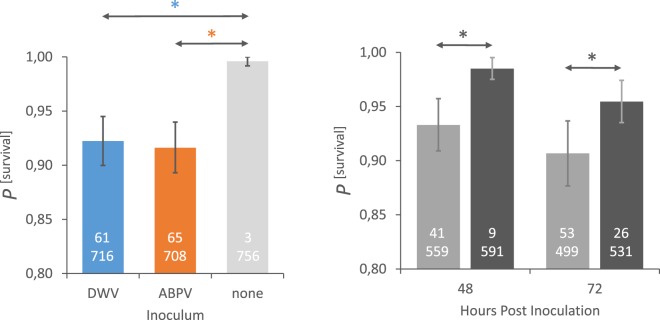
Left panel: The predicted marginal mean and 95% confidence intervals across all sampling time points, colonies and populations of the probability of survival for the adult bees inoculated with either DWV (blue), ABPV (orange) or uninoculated (grey). Right panel: The predicted marginal mean and 95% confidence intervals across all virus inoculation trials of the probability of survival for the adult bees from either the MS (light grey) or MR (dark grey) colonies, at two different time-points post-inoculation. Statistically significant differences are marked with an asterisk. The absolute numbers of dead (top number) and live bees (bottom number) involved in the estimations are shown in each column.

The only aspect in these virus experiments where there is a clear difference between the MR and the MS bees is in the survival rates of the virus-inoculated adult bees (Fig. 3; Table 3; Table S2). In both DWV and ABPV inoculated bees there is a significantly higher survival rate for MR bees than for MS bees, both at 48 hpi and 72 hpi (Fig. 3). There is little difference in survival probability between bees inoculated with either DWV or ABPV across both honeybee populations combined (Fig. 3), but a very clear reduction in the survival probability of DWV/ABPV-inoculated bees compared to uninoculated control bees (Fig. 3).

**Table 3:** Analysis-of-deviance tables (Type III test) from generalized linear mixed models that investigate the effect of colonies history (MR, MS), treatment (DWV, ABPV), and time (48, 72, 120 hours post inoculation) on the survival probability of adults bees. Non-significant terms (italicized) were removed stepwise until the final minimal adequate model starting from the bottom row.

Model ID	Life stage	Response Variable	Explanatory Variables	Χ^2^	Df	AICc	con.R^2^	p-value
9	Adults	Survival (alive/dead)	HPI	9.50	1	152.48	0.45	<0.01
			Treatment	28.86	2			<0.001
			Colony history	16.53	1			<0.001
			*Colony history × HPI*	*3*.*11*	*1*	*152*.*09*	*0*.*46*	*0*.*07*
			*Colony history × Treatment*	*0*.*01*	*2*	*156*.*53*	*0*.*96*	*0*.*99*
			*HPI × Treatment*	*0*.*79*	*2*	*162*.*65*	*0*.*96*	*0*.*67*
			*Colony history × HPI × Treatment*	*0*.*60*	*2*	*169*.*85*	*0*.*96*	*0*.*73*

## Discussion

The main result of these experiments is that there is no difference in susceptibility to DWV or ABPV infection between bees from the MR population and those from the MS population, for both adult bees and larvae. However, there is a significantly lower mortality for virus-infected adults from the MR population, relative to virus-infected adults from the MS population, with no major differences between ABPV-infected or DWV-infected bees. There was no difference in mortality between uninfected bees from the MR or MS populations. Survivability in larvae was not possible to assess since the experimental virus dose was optimized to prevent larvae from dying so that infection dynamics could be monitored and any differences in virus susceptibility could be observed.

Another major result from these experiments is that oral inoculation with DWV has no effect on the background infection dynamics of ABPV and *vice versa*, for both larvae and adults, *i*.*e*. the viruses did not appear to compete or interfere with each other. This supports earlier research showing that different bee viruses evoke entirely different transcriptional responses and biological processes after oral inoculation^26^, and that different viruses develop equally well in isolation or when co-inoculated with other viruses^26^. Many bee viruses also show high tissue-specificity^27^, such that any temporal-spatial isolation of the viruses reduces direct competition at the molecular level. Possible genetic changes to the inoculated viruses during the time course as well as any molecular interactions with the host or other inoculated or background viruses are the subject of ongoing investigations.

The final major result is the shape of the infection curves of the uninoculated/alternate virus-inoculated controls. These are very different for larvae and adults, and also slightly different for DWV- or ABPV-inoculated bees, but are highly similar for the different populations and between the uninoculated and alternate virus-inoculated groups, comprising furthermore data from many different colonies. Although there was variation across the time-course in the mRNA expression levels of our internal reference gene RP49, these were insignificant compared to the logarithmic-scale variation in the virus titres across the same time-frame, and were therefore not responsible for the shapes of the curves. Since these represent the infections in the least manipulated bees, they are of special interest for identifying the natural interaction between viruses and their host. We have currently no explanation for why the natural DWV and ABPV titres in larvae should go down, and then up, or why in adult bees they should go up, then down, before levelling off. This material is currently the subject of further investigation. One final point that requires clarification is the relatively high initial DWV titre in newly hatched adult bees of both the MR and MS populations, or for that matter any background DWV and ABPV at all, given that the original colonies were sourced from varroa-free Åland^28,29^ and were presumably DWV- and ABPV-free, given Ålands virus-free history (Doublet *et al*. *unpublished data*). Since the 0 hpi material never was exposed to virus in the laboratory, our best explanation is that these colonies became contaminated during the 2 months that were needed to turn over the bee populations after introduction of the MR and MS queens. Given their origins^23,30^, these queens will undoubtedly have been infected with at least DWV, which is efficiently transmitted through the eggs to the resulting progeny^12,13,31^, as well as to the rest of the colony through larval care and social interactions^32,33^. However, any virus infections thus acquired would be similar for all bees and experimental groups in our study and part of the molecular background to which the experimental inoculations are applied. DWV-symptomatic adult bees were not observed during the experiments, since DWV symptoms develop during the pupal phase, generally due to transmission by varroa. However, some of the adult bees that were orally inoculated with APBV were observed to be trembling, which is a symptom of APBV infection.

Differences in DWV virulence have been associated with different major DWV genotypes, with the DWV-A genotype being more wide-spread but less virulent than the DWV-B genotype^34^. Laboratory experiments have shown that the DWV-B genotype is preferentially transmitted by varroa and through injection compared to the DWV-A genotype^35^. The persistence of both strains in natural colonies attests to the importance of other transmission routes, such as oral and sexual transmission in maintaining this balance^13,36^, together with the different consequences of virulence at individual bee and colony level. Similar differences in virulence and infectivity have also been described for the members of the ABPV-complex (ABPV, KBV, IAPV) in relation to the various transmission routes and different consequences for individual and colony-level mortality^10^. Although we cannot rule out possible interactions of our inoculated viruses with background virus infections, or the host molecular condition (which will be the subject of a separate investigation), the focus of this study is how these experimental virus infections differentially affect bees from MR and MS colonies, both through the infection time-course and through adult mortality.

The enhanced survival of virus-infected adult bees from the Gotland MR population, relative to those of the MS population, despite near identical susceptibility to virus infections across the DWV and ABPV infection time-courses, suggests that host tolerance, rather than resistance, is an important component of the naturally adapted survival mechanisms of this population and that their individual-level tolerance response to virus infections complements earlier work demonstrating a colony-level tolerance to DWV^23^. In animal health, tolerance is a highly effective mechanistic response to disease but is often overlooked, especially when resistance is present^25^. Tolerance adaptations do not inflict harm on the parasite, unlike resistance, so it is expected to fix in the population rather than causing an open-ended antagonistic coevolution, as is the case with resistance evolution^25^. This tolerance to virus infections has been demonstrated on earlier generations within the Gotland MR honeybee population and in different geographical locations^23^, so it appears to have a genetic heritable component to its expression. It is unclear if there are host fitness costs associated with this virus tolerance and to what degree this virus tolerance has on the overall long-term survival of the population in relation to the resistant mechanisms that limit the population growth of varroa mites.

The honeybee host has a unique social organization that presents its own complexity in this system with multiple levels of possible host-parasite interactions and adaptation to parasites and disease infections. Resistance at the individual level could translate to tolerance at the colony level and *vice versa*^37^. For example, despite the individual level mite resistance of inhibiting mite reproduction, colony level varroa infestation rates in the Gotland honeybee population can still be high^22,23^, suggesting other factors could contribute to the population’s natural long-term survival. The results of this study demonstrate that tolerance rather than resistance, plays an important role in defence against varroa-vectored virus infections, at both levels of colony organization for this population^23^. In contrast to our study, reduced susceptibility to virus infections has been observed in savannah honeybees in Africa^38^ and in selectively bred mite-resistant honeybees, compared to non-selected lines, in the U.S.^39^. However, these studies did not exclude confounding influences on virus infections by the virus-vectoring varroa mite, which likely could have impacted their results. The disparity between these studies only demonstrates the potential for varying adaptive responses of honeybee populations to virus infections, which would be expected under varying environmental conditions^40,41^. Current efforts are underway to explore virus resistance and tolerance defence mechanism in other unique naturally adapted mite-resistant populations and to assess the potential to include virus response mechanisms in breeding programs to improve bee health and sustainable beekeeping.

In conclusion, individuals from the Gotland MR honeybee populations survive better than MS honeybees having a higher threshold for virus infections before bee health is compromised. This elevated virus tolerance for MR bees at the individual level supports the previously inferred virus tolerance at colony level^23,30^, but excluding the confounding influences of varroa mite infestation and environmental factors *e*.*g*. the different colony development dynamics between the MR and MS bees^22^. Moreover, the observed virus tolerance applies equally and similarly to DWV- or ABPV- infected bees, suggesting that the tolerance mechanism may be primarily generic, rather than virus specific.

## Materials and Methods

### Origin and management of honeybee colonies

The mite-resistant (MR) honeybees originated from an isolated population on the island of Gotland, Sweden that has been surviving without mite control treatments since 1999. This population was established as a selection experiment on honeybee survival with a new invasive parasite^21^ and has demonstrated a variety of adaptations that enable their long-term survival with varroa mite infestation^22,23^. The mite-susceptible (MS) honeybees originated from a local unselected honeybee population near Uppsala that required regular varroa mite control interventions to avoid colony death. Six open-mated queens from each population were established in small 6-frame experimental colonies stocked with equal amounts of honeybees from Åland, a group of islands between Sweden and Finland. Åland is still entirely free from varroa mites^28,29^ and have undetectable levels of both DWV and ABPV in general surveys (Doublet *et al*. *unpublished data*) so these bees provided the ideal experimental background for virus infection experiments, having never been exposed to the virus or the vector. Experiments did not begin until all individuals in each colony were the offspring of the introduced queens; approximately 2 months after the queens were introduced.

### Preparation and optimization of virus material

The DWV and ABPV inocula for the oral infection experiments were prepared by propagating reference DWV-A and ABPV virus stocks each in fifty white eyed pupae from the varroa-free colonies from Åland (see above). Each pupae was injected with 1 micro litre of a 1/10000 dilution of purified concentrated virus stock (equivalent to about 10^3^ virus genome copies/bee), according to standard pupal propagation procedures^15^. From these 50 pupae, a clarified crude extract was made by homogenizing the pupae in a blender with 10 mL 0.5 M Phophate Buffer, pH 8.0 (DWV) or 10 mL 0.01 M Phosphate Buffer, pH 7.0 (ABPV),and stored in 50 μl aliquots at −80 °C^15^. These crude extracts were used for the virus infection experiments. The virological composition of the propagated virus stocks was determined using RT-qPCR assays for seven common bee viruses that can be propagated through injection^13^: DWV-A, DWV-B, ABPV, IAPV, KBV, *sacbrood virus* (SBV) and *black queen cell virus* (BQCV). Using an MuMLV 1^st^ strand cDNA kit (ThermoFisher, Waltham, MA, USA), random hexamer-primed cDNA was prepared from 1 ug of RNA extracted from the propagated inoculum to which 25 ng of RNA250 (ThermoFisher, Waltham, MA, USA) was added, as an exogenous reference RNA for normalizing the relative titres of the viruses against a common fixed standard. A six-step 10-fold dilution series was made from each cDNA and used as templates for the virus-specific qPCR reactions. The dilution series were used to obtain the reaction efficiencies of the different assays. These reaction efficiencies were then used to estimate the relative amount of each virus in each cDNA dilution in relation to the common fixed RNA250 standard^42^. The average of these multiple estimates of the relative amounts of each virus in each inoculum was used to construct the pie-charts in Supplementary Fig. S3.

The infectivity of these crude extracts was tested in optimization experiments to identify the optimum dose for experimentation, using larval and adult mortality rates as the optimization criteria. The optimum virus dose was defined as the highest single virus dose that did not cause larvae or adult bee mortality before 96 hours post inoculation (hpi). This dose selection criteria was used so that early (non-lethal) virus infectivity dynamics could be studied, as well as possible subsequent differential mortality between mite-resistant and mite-susceptible bees. The optimum single inoculation dose for larvae was 1.5 ± × 10^8^ DWV genome equivalents and 5.4 ± × 10^7^ ABPV genome equivalents, and for adults 6.0 ± × 10^8^ DWV genome equivalents and 2.1 ± × 10^8^ ABPV equivalents, as determined by RT-qPCR analysis of the crude extracts. These levels are consistent with previous estimates of the infectious doses for these viruses^15,17,43,44^.

### Experimental design

The infection experiments were conducted separately on newly emerged adult bees and on newly hatched larvae. Each infection experiment consisted of several replicate infection trials for each MR and MS colony included in the experiment (Table S3). Where possible, the same MR and MS colonies were used for both the larval and adult experiments. Each infection trial consisted of one cohort of DWV inoculated bees, one cohort of ABPV inoculated bees and one cohort of uninoculated control bees. The inoculation strategy consisted of bees fed with a single infectious dose for a short period followed by non-contaminated food for the remainder of the time course. This infection strategy ensures that any increase in virus titres through the time course represents a newly established infection, rather than passive accumulation of virus inoculum, as would be the case with continuous inoculation. From each cohort of bees in each infection trial, adult bees were sampled at 0, 12, 24, 48, 72 and 120 hours post inoculation (hpi) and larvae were sampled at 0, 24, 72 and 120 hpi, representing the time-course. The experiments were conducted during July-August 2016 (larvae) and August-September 2016 (adults).

### *In-vitro* larval infection experiments

The larval infection experiments were conducted on larvae from 4 MR colonies and 4 MS colonies. Each infection trial was replicated at least two times, depending on the availability of larvae from the colony concerned. Larvae of similar age were obtained by confining the queens of the experimental colonies to a single frame for 24 hours for egg-laying. First instar larvae (between 24–36 hours old) were grafted with a Chinese grafting tool (Bienenzuchtgeräte, Graze, Weinstadt, Germany) into individual wells of 48-well tissue culture plates (Falcon™ Polystyrene Microplates), each containing 10 μL of pre-warmed larval food consisting of 50% royal jelly (Vceli Produkty, Nedasov, Czech Republic), and 50% aqueous solution of D-glucose (12%), D-fructose (12%), and yeast extract (2%)^45^, following standard larval rearing methods^46^. Extra larvae were grafted in each cell to compensate for any mortality due to the grafting procedure. The larvae were pre-incubated for 24 hours at 35 °C with a relative humidity of 96%, after which all dead and excess larvae were removed, such that 48 living larvae, one per well, were retained for the infection experiment. Larval mortality due to grafting procedure generally did not exceed 20%. The viable larvae (approximately 48 hour old) were then fed with larval food. For the larvae cohorts to be inoculated with virus, the larval food was spiked with the optimum single infectious dose of DWV or ABPV, as determined above. The larvae were fed daily according to established protocols^46^ and any dead larvae were removed. On each sampling occasion (see above), 4 live larvae from each infection cohort were collected in microcentrifuge tubes and stored at −20 °C until further analysis^47^.

### Adult bee cage infection experiments

The adult infection experiments were conducted on newly emerged adult bees from 5 MR colonies and 3 MS colonies. Each infection trial was replicated between 1–4 times, depending on the availability of newly hatched adult bees from the colony concerned. The bees were hatched on caged frames inside an incubator at a constant 35 °C temperature and 96% relative humidity^48^. For each inoculation cohort fifty newly emerged adults from each colony were placed in separate Lyson queen cages (Łyson, Klecza Dolna, Poland), and fed over a 24-hour period the optimum DWV and ABPV inoculation dose (as described above) in 2 mL Bifor^®^, a 66% w/w commercial honeybee sugar solution with a 2/1/1 ratio of sucrose/fructose/glucose (Nordic Sugar A/S, Copenhagen, Denmark) with control bees receiving just Bifor^®^. After inoculation, all cohorts of bees were fed uncontaminated Bifor^®^ *ad libitum* for the remainder of the time-course. On each sampling occasion (see above) and for each infection cohort, all dead bees were counted and removed, retaining 5 bees for analysis, while 5 live bees were also sampled. Both live and dead bee samples were stored at −20 °C until further analysis.

### Sample processing and RT-qPCR assays

Each experimental time-course sample, containing either 4 larvae or 5 adult bees, was placed in a mesh bag and ground to powder using liquid nitrogen and a pestle. A primary homogenate was produced by adding 200 μl/bee sterile water to each ground sample and mixing vigorously^47^. Total RNA was extracted from 100 μl of this homogenate by a QiaCube robot following the RNAeasy protocol for plants (Qiagen). The RNA was eluted in 50-μl RNase-free water, the RNA concentration was estimated by NanoDrop and the purified RNA was stored at −80 °C until further processing.

The amounts of DWV and ABPV RNA, as well as RP49 mRNA (a honeybee internal reference gene commonly used for normalizing between-sample differences in RNA quantity and quality^15^) were determined using reverse transcription quantitative PCR (RT-qPCR), using the iScript One Step RT-PCR kit (Bio-Rad) with SYBR Green as the detection chemistry and the Bio-Rad CFX connect thermocycler. The reactions were performed in 20 μl volumes containing 0.2 μM of the forward and the reverse primers (Table S4), 3 μl RNA, 10 μl SYBR Green RTmix and 0.4 μl of iScript reverse transcriptase. The thermocycling profile for both assays was as followed: 10 min at 50 °C for cDNA synthesis, 5 min at 95 °C for inactivation of the reverse transcriptase following 40 cycles of 10 sec. at 95 °C for denaturation and 30 sec. at 58 °C for annealing/extension and data collection. Amplification was followed immediately by a Melting Curve analysis to confirm the identity of the amplification products, by incubating at 60 sec: 95 °C, 60 sec. 65 °C and fluorescence reading at 0.5 °C increments between 65 °C and 95 °C. For each assay, a 10-fold serial dilution series of a positive cloned (plasmid) control of known concentration was also run on each reaction plate, as well as a negative (water) control, to establish the calibration curves for absolute quantification, as performed by the BioRad CFX software. All positive PCR amplifications were checked for product identity through visual inspection of their characteristic Melting Curves, prior to inclusion in the quantitative analyses.

### Data conversion and statistical analyses

For each individual sample, the raw DWV and ABPV titres were divided by the specific estimated amount of RP49 mRNA in the sample and multiplied by the average amount of RP49 mRNA for the entire experiments. This was done for the larval or adult infection respectively in order to normalize the data for sample-specific differences in RNA quality and quantity^49,50^. Across the entire experiment, there was no consistent relationship between the observed virus titres and the reference RP49 mRNA levels, as determined by regression analyses, validating the use of RP49 mRNA for normalising virus titres (Fig. S4). This value was then multiplied by the various dilution factors incurred during processing and assaying to obtain the estimated normalized DWV and ABPV titres per individual bee. These normalized DWV and ABPV titres over time ranged over five (DWV) and eight (ABPV) orders of magnitude, which is normal for the growth phase of a rapidly replicating (viral) pathogen^15^. The normalized virus titres were analysed with linear mixed models using the R software^51^ and the package *lme4*. Colony history (MR or MS), the treatment (inoculated or uninoculated, with the respective virus), and the hours post inoculation (hpi) were included as categorical fixed effects. Time was used as categorical variable in order to perform pair wise comparisons and because titres did not simply increase/decrease over the course of the experiments. To account for the hierarchical data structure (multiple samples of a colony), sample was included as a random effect nested within each colony. The Type III test was used in the analysis since the sampling was unbalanced (different number of colonies with different numbers of replications within colonies). In order to obtain normally distributed residuals (checked visually), the function *transformTukey* was used from the package *rcompanion*, which loops over exponents that maximizes/minimizes the Shapiro-Wilks/Anderson-Darling statistic. After performing the statistic with the recommended transformation (Table 1) the values were back-transformed to original scale and then transformed to log10 scale for illustrative purposes. Because the zero time point virus titres were taken before the assignment to the treatments they were analysed separately with the colony history as fixed and the colony as random effects.

The relationship between the number of dead and alive adult bees was used to calculate the survival probability using a binomial likelihood (logit link function) and a generalized linear mixed model using the package *lme4*. Scaled residuals were checked using the package DHARMa. We tested if the colony history, inoculation with either virus, or the time after inoculation interactively affected the survival probability.

For all models, the type III test with function *Anova* from the package *car* was used to stepwise remove non-significant variables until the minimal adequate model was obtained. Tukey method adjusted pair wise comparisons (also on models that still included interactions of interest) were performed using the package *multcomp*. To obtain predicted marginal means from each model we used the packages *lsmeans*. Further we calculated the conditional R^2^ value, which accounts for the fixed and random model part, and the second-order AIC.

## Supplementary information

Thaduri et al. 2019. Supplementary Figures and Tables

## Data Availability

The datasets generated during and/or analysed during the current study are available from the corresponding author on reasonable request.

## References

[1] Rundlöf M (2015). Seed coating with a neonicotinoid insecticide negatively affects wild bees. Nature.

[2] Osterman J (2019). Clothianidin seed-treatment has no detectable negative impact on honeybee colonies and their pathogens. Nat. Commun..

[3] Boecking O, Genersch E (2008). Varroosis - The ongoing crisis in bee keeping. J. fur Verbraucherschutz und Leb..

[4] Le Conte, Y., Ellis, M. & Ritter, W. Varroa mites and honey bee health: can Varroa explain part of the colony losses? *Apidologie* **41** (2010).

[5] Schroeder DC, Martin SJ (2012). Deformed wing virus. Virulence.

[6] Mordecai GJ, Wilfert L, Martin SJ, Jones IM, Schroeder DC (2016). Diversity in a honey bee pathogen: first report of a third master variant of the Deformed Wing Virus quasispecies. ISME J..

[7] de Miranda JR, Cordoni G, Budge G (2010). The Acute bee paralysis virus–Kashmir bee virus–Israeli acute paralysis virus complex. J. Invertebr. Pathol..

[8] Martin Stephen J. (2002). The role of Varroa and viral pathogens in the collapse of honeybee colonies: a modelling approach. Journal of Applied Ecology.

[9] Wilfert L (2016). Deformed wing virus is a recent global epidemic in honeybees driven by Varroa mites. Science (80-.)..

[10] de Miranda JR, Genersch E (2010). Deformed wing virus. J. Invertebr. Pathol..

[11] Bowen-Walker PL, Martin SJ, Gunn A (1999). The transmission of deformed wing virus between honeybees (Apis mellifera L.) by the ectoparasitic mite Varroa jacobsoni Oud. J. Invertebr. Pathol..

[12] Yue, C., Schroeder, M., Gisder, S. & Genersch, E. Vertical-transmission routes for deformed wing virus of honeybees (Apis mellifera). *J*. *Gen*. *Virol*. **88** (2007). 10.1099/vir.0.83101-0 17622639

[13] de Miranda JR, Fries I (2008). Venereal and vertical transmission of deformed wing virus in honeybees (Apis mellifera L.). J. Invertebr. Pathol..

[14] Genersch, E. & Aubert, M. Emerging and re-emerging viruses of the honey bee (Apis mellifera L). *Vet*. *Res*. **41** (2010). 10.1051/vetres/2010027 PMC2883145 20423694

[15] de Miranda JR (2013). Standard methods for virus research in Apis mellifera. J. Apic. Res..

[16] Amiri E (2018). Quantitative patterns of vertical transmission of deformed wing virus in honey bees. PLoS One.

[17] Moeckel, N., Gisder, S. & Genersch, E. Horizontal transmission of deformed wing virus: pathological consequences in adult bees (Apis mellifera) depend on the transmission route. *J*. *Gen*. *Virol*. **92** (2011). 10.1099/vir.0.025940-0 20965988

[18] Locke B (2016). Natural Varroa mite-surviving Apis mellifera honeybee populations. Apidologie.

[19] Rosenkranz P, Aumeier P, Ziegelmann B (2010). Biology and control of Varroa destructor. J. Invertebr. Pathol..

[20] Oddie M (2018). Rapid parallel evolution overcomes global honey bee parasite. Sci. Rep..

[21] Fries I, Imdorf A, Rosenkranz P (2006). Survival of mite infested (Varroa destructor) honey bee (Apis mellifera) colonies in a Nordic climate. Apidologie.

[22] Locke B, Fries I (2011). Characteristics of honey bee colonies (Apis mellifera) in Sweden surviving Varroa destructor infestation. Apidologie.

[23] Locke B, Forsgren E, de Miranda JR (2014). Increased Tolerance and Resistance to Virus Infections: A Possible Factor in the Survival of Varroa destructor-Resistant Honey Bees (Apis mellifera). PLoS One.

[24] Locke B (2016). Inheritance of reduced Varroa mite reproductive success in reciprocal crosses of mite-resistant and mite-susceptible honey bees (Apis mellifera). Apidologie.

[25] Råberg L, Graham AL, Read AF (2009). Decomposing health: tolerance and resistance to parasites in animals. Philos. Trans. R. Soc. B.

[26] Ryabov EV, Fannon JM, Moore JD, Wood GR, Evans DJ (2016). The Iflaviruses Sacbrood virus and Deformed wing virus evoke different transcriptional responses in the honeybee which may facilitate their horizontal or vertical transmission. PeerJ.

[27] Fievet J (2006). Localization of deformed wing virus infection in queen and drone Apis mellifera L. Virol. J..

[28] European Commission. Commission Implementing Decision of 11 October 2013 recognising parts of the Union as free from varroosis in bees and establishing additional guarantees required in intra-Union trade and imports for the protection of their varroosis-free status. *Off*. *J*. *Eur*. *Union* **273** (2013).

[29] More, S. *et al*. Assessment of listing and categorisation of animal diseases within the framework of the Animal Health Law (Regulation (EU) No 2016/429): infestation with Varroa spp. (varroosis). *EFSA J*. **15** (2017). 10.2903/j.efsa.2017.4997 PMC7009930 32625294

[30] Thaduri S, Locke B, Granberg F, de Miranda JR (2018). Temporal changes in the viromes of Swedish Varroa-resistant and Varroa-susceptible honeybee populations. PLoS One.

[31] Amiri E, Meixner MD, Kryger P (2016). Deformed wing virus can be transmitted during natural mating in honey bees and infect the queens. Sci. Rep..

[32] Yue C, Genersch E (2005). RT-PCR analysis of Deformed wing virus in honeybees (Apis mellifera) and mites (Varroa destructor). J. Gen. Virol..

[33] Chen Y, Evans J, Feldlaufer M (2006). Horizontal and vertical transmission of viruses in the honey bee, Apis mellifera. J. Invertebr. Pathol..

[34] McMahon DP (2016). Elevated virulence of an emerging viral genotype as a driver of honeybee loss. Proc. R. Soc. B Biol. Sci..

[35] Ryabov EV (2014). A virulent strain of deformed wing virus (DWV) of honeybees (Apis mellifera) prevails after Varroa destructor-mediated, or *in vitro*, transmission. PLoS Pathog..

[36] Locke B, Semberg E, Forsgren E, de Miranda JR (2017). Persistence of subclinical deformed wing virus infections in honeybees following Varroa mite removal and a bee population turnover. PLoS One.

[37] Kurze C, Routtu J, Moritz RFA (2016). Parasite resistance and tolerance in honeybees at the individual and social level. Zoology.

[38] Strauss, U., Human, H., Gauthier, L., Crewe, R. & Dietemann, V. Seasonal prevalence of pathogens and parasites in the savannah honeybee (Apis mellifera scutellata). *J*. *Invertebr*. *Pathol*. **114** (2013). 10.1016/j.jip.2013.05.003 23702244

[39] Khongphinitbunjong K (2016). Responses of Varroa -resistant honey bees (Apis mellifera L.) to Deformed wing virus. J. Asia. Pac. Entomol..

[40] Locke B, Le Conte Y, Crauser D, Fries I (2012). Host adaptations reduce the reproductive success of Varroa destructor in two distinct European honey bee populations. Ecol. Evol..

[41] Thompson JN (1999). The evolution of species interactions. Science.

[42] Pfaffl MW, Horgan GW, Dempfle L (2002). Relative expression software tool (REST(C)) for group-wise comparison and statistical analysis of relative expression results in real-time PCR. Nucleic Acids Res..

[43] Meeus I, de Miranda JR, de Graaf DC, Wäckers F, Smagghe G (2014). Effect of oral infection with Kashmir bee virus and Israeli acute paralysis virus on bumblebee (Bombus terrestris) reproductive success. J. Invertebr. Pathol..

[44] Carrillo-Tripp J (2016). *In vivo* and *in vitro* infection dynamics of honey bee viruses. Sci. Rep..

[45] Aupinel P (2005). Improvement of artificial feeding in a standard *in vitro* method for rearing Apis mellifera larvae. Bull. Insectology.

[46] Crailsheim K (2013). Standard methods for artificial rearing of Apis mellifera larvae. J. Apic. Res..

[47] Forsgren E, Locke B, Semberg E, Laugen AT, Miranda JRde (2017). Sample preservation, transport and processing strategies for honeybee RNA extraction: Influence on RNA yield, quality, target quantification and data normalization. J. Virol. Methods.

[48] Williams GR (2013). Standard methods for maintaining adult Apis mellifera in cages under *in vitro* laboratory conditions. J. Apic. Res..

[49] Locke B, Forsgren E, Fries I, de Miranda JR (2012). Acaricide treatment affects viral dynamics in Varroa destructor-infested honey bee colonies via both host physiology and mite control. Appl. Environ. Microbiol..

[50] Lourenco AP, Mackert A, Cristino A, dos S, Simoes ZLP (2008). Validation of reference genes for gene expression studies in the honey bee, Apis mellifera, by quantitative real-time RT-PCR. Apidologie.

[51] R Core Team. R: A language and environment for statistical computing (2017).

