# Supplementary material for: Disentangling host-parasite-pathogen interactions in a varroa-resistant honeybee population reveals virus tolerance as an independent, naturally adapted survival mechanism
Source: Sci Rep. 2019 Apr 17;9:6221. doi: 10.1038/s41598-019-42741-6 (PMC6470206; doi:10.1038/s41598-019-42741-6)
Supplement: Supplementary file 1 — Thaduri et al. 2019. Supplementary Figures and Tables [file 41598_2019_42741_MOESM1_ESM.pdf]

# Disentangling host-parasite-pathogen interactions in a varroa-resistant honeybee population reveals virus tolerance as an independent, naturally adapted survival mechanism

Srinivas THADURI[1], Jörg G. STEPHAN[1] Joachim R. de MIRANDA[1] and Barbara LOCKE[1]*

[1]Department of Ecology, Swedish University of Agricultural Sciences, 75007 Uppsala

srinivas.thaduri@slu.se; jorg.stephan@slu.se; joachim.de.miranda@slu.se; barbara.locke@slu.se

*Correspondence: barbara.locke@slu.se; Tel.: +46-18-672-339

## SUPPLEMENTARY FILES

**Table S1: Effect of co-infection on DWV and ABPV titres.** Analysis-of-deviance tables (Type III test) from linear mixed models investigating the effect of the natural background infection level of the other virus on the titres of the infected virus, respectively. Replicate nested within colony ID was included in all models as random effect.

| Life stage | Treatment | Response Variable | Explanatory Variables | $X^2$ | Df | AICc | con.$R^2$ | p-value |
|---|---|---|---|---|---|---|---|---|
| Adult | DWV (MS+MR) | $DWV^{0.425}$ | ABPV | 0.85 | 1 | 1031 | 0.40 | 0.35 |
| | ABPV (MS+MR) | Log(ABPV+1) | DWV | 0.33 | 1 | 224 | 0.78 | 0.56 |
| | ABPV (MS+MR) | $DWV^{0.1}$ | ABPV | 5.03 | 1 | 316 | 0.74 | **0.02** |
| | DWV (MS+MR) | $ABPV^{0.125}$ | DWV | 0.004 | 1 | 285 | 0.25 | 0.94 |
| Larvae | DWV (MS+MR) | $DWV^{-0.225}$ | ABPV | 1.22 | 1 | -235 | 0.49 | 0.26 |
| | ABPV (MS+MR) | $ABPV^{-0.825}$ | DWV | 0.01 | 1 | -1178 | 0.00 | 0.90 |
| | ABPV (MS+MR) | $DWV^{0.15}$ | ABPV | 2.07 | 1 | 241 | 0.25 | 0.14 |
| | DWV (MS+MR) | $ABPV^{0.375}$ | DWV | 0.02 | 1 | 395 | 0.31 | 0.86 |

**Table S2: Absolute numbers of survival in the adult bee experiments.**

| Colony history | HPI | Treatment | Dead | Alive |
|---|---|---|---|---|
| MR | 48h | ABPV | 4 | 196 |
| MR | 48h | DWV | 5 | 195 |
| MR | 48h | NoVirus | 0 | 200 |
| MR | 72h | ABPV | 14 | 176 |
| MR | 72h | DWV | 12 | 175 |
| MR | 72h | NoVirus | 0 | 180 |
| MS | 48h | ABPV | 22 | 178 |
| MS | 48h | DWV | 17 | 183 |
| MS | 48h | NoVirus | 2 | 198 |
| MS | 72h | ABPV | 25 | 158 |
| MS | 72h | DWV | 27 | 163 |
| MS | 72h | NoVirus | 1 | 178 |

**Table S3: Experimental design.** Visual representation of the design of the virus infection experiments in larvae and adults. Shown are the population (POP) of origin (MR or MS); the name of the colony (COL), the experimental replicate (REP) for each colony and the samples collected at the different time points for uninoculated, DWV-inoculated and ABPV-inoculated bees (shaded fields), with the live samples indicated by a 'Θ' above the diagonal and the dead samples indicated by a '†' below the diagonal.

**LARVAE**

| POP | COL | REP | NONE 0 hr | NONE 12 hr | NONE 24 hr | NONE 48 hr | NONE 72 hr | NONE 120 hr | DWV 12 hr | DWV 24 hr | DWV 48 hr | DWV 72 hr | DWV 120 hr | ABPV 12 hr | ABPV 24 hr | ABPV 48 hr | ABPV 72 hr | ABPV 120 hr |
|---|---|---|---|---|---|---|---|---|---|---|---|---|---|---|---|---|---|---|
| MR | K4 | 1 | Θ | | | Θ | Θ | Θ | | | Θ | Θ | Θ | | | Θ | Θ | Θ |
| | K5 | 1 | Θ | | | Θ | Θ | Θ | | | Θ | Θ | Θ | | | Θ | Θ | Θ |
| | | 2 | Θ | | | Θ | Θ | Θ | | | Θ | Θ | Θ | | | Θ | Θ | Θ |
| | K8 | 1 | | | | Θ | Θ | Θ | | | Θ | Θ | Θ | | | Θ | Θ | Θ |
| | | 2 | Θ | | | Θ | Θ | | | | Θ | Θ | | | | Θ | Θ | |
| | K9 | 1 | | | | Θ | Θ | Θ | | | Θ | Θ | Θ | | | Θ | Θ | Θ |
| | | 2 | Θ | | | Θ | Θ | Θ | | | Θ | Θ | Θ | | | Θ | Θ | Θ |
| MS | K1 | 1 | Θ | | | Θ | Θ | Θ | | | Θ | Θ | Θ | | | Θ | Θ | Θ |
| | | 2 | Θ | | | Θ | Θ | | | | Θ | Θ | | | | Θ | Θ | |
| | K2 | 1 | Θ | | | Θ | Θ | Θ | | | Θ | Θ | Θ | | | Θ | Θ | Θ |
| | | 2 | Θ | | | Θ | Θ | Θ | | | Θ | Θ | Θ | | | Θ | Θ | Θ |
| | K6 | 1 | Θ | | | Θ | Θ | Θ | | | Θ | Θ | Θ | | | Θ | Θ | Θ |
| | | 2 | Θ | | | Θ | Θ | Θ | | | Θ | Θ | Θ | | | Θ | Θ | Θ |
| | K7 | 1 | | | | Θ | Θ | Θ | | | Θ | Θ | Θ | | | Θ | Θ | Θ |

**ADULTS**

| POP | COL | REP | NONE 0 hr | NONE 12 hr | NONE 24 hr | NONE 48 hr | NONE 72 hr | NONE 120 hr | DWV 12 hr | DWV 24 hr | DWV 48 hr | DWV 72 hr | DWV 120 hr | ABPV 12 hr | ABPV 24 hr | ABPV 48 hr | ABPV 72 hr | ABPV 120 hr |
|---|---|---|---|---|---|---|---|---|---|---|---|---|---|---|---|---|---|---|
| MR | B_nuc | 1 | Θ | | | Θ | Θ | Θ | | | Θ | Θ / † | Θ / † | | | Θ | Θ | Θ |
| | | 2 | Θ | Θ | Θ | Θ | Θ | Θ | Θ | Θ | Θ | Θ | Θ / † | Θ | Θ | Θ / † | Θ | Θ |
| | K5 | 1 | Θ | Θ | Θ | Θ | Θ | Θ | Θ | Θ | Θ | Θ | Θ / † | Θ | Θ | Θ | Θ | Θ / † |
| | K8 | 1 | Θ | | | Θ | Θ | Θ | | | Θ | Θ / † | Θ | | | Θ | Θ | Θ |
| | K9 | 1 | Θ | | | Θ | Θ | Θ | | | Θ | Θ / † | Θ / † | | | Θ | Θ / † | Θ / † |
| | K5_mix | 1 | Θ | Θ | Θ | Θ / † | Θ | Θ | Θ | Θ | Θ / † | Θ | Θ / † | Θ | Θ | Θ / † | Θ | Θ / † |
| MS | C_nuc | 1 | Θ | Θ | Θ | Θ / † | Θ | Θ | Θ | Θ | Θ / † | Θ | Θ / † | Θ | Θ | Θ / † | Θ | Θ / † |
| | K2 | 1 | Θ | | | Θ | Θ | Θ | | | Θ | Θ / † | Θ / † | | | Θ / † | Θ / † | Θ / † |
| | | 2 | Θ | | | Θ | | Θ | | | Θ / † | | Θ / † | | | Θ / † | | Θ / † |
| | | 3 | Θ | Θ | Θ | Θ | Θ | Θ | Θ | Θ | Θ / † | Θ | | Θ | Θ | Θ / † | Θ | Θ / † |
| | | 4 | Θ | Θ | Θ | Θ | Θ | Θ | Θ | Θ | Θ / † | Θ | Θ / † | Θ | Θ | Θ / † | Θ | Θ / † |
| | K6 | 1 | Θ | | | Θ | Θ | Θ | | | Θ / † | Θ / † | Θ | | | Θ / † | Θ / † | Θ |

**Table S4. RT-qPCR assays**. Details of the RT-qPCR assays used in the experiments, including the target name, the sequences of the forward and reverse primers, the product size, the reaction efficiency (*E*), linearity of the dilution standards ($r^2$) and the melting temperature of the reaction products ($T_m$).

| Assay | Primers | Sequence (5' - 3') | Size (nt) | *E* | $r^2$ | $T_m$ |
|---|---|---|---|---|---|---|
| DWV-A/B | DWV-F1425 | CGTCGGCCTATCAAAG | 417 | 1.681 | 0.997 | 79.5 ºC |
| | DWV-B1806 | CTTTTCTAATTCAACTTCACC | | | | |
| DWV-B | VaDV-F1409 | GCCCTGTTCAAGAACATG | 413 | 1.723 | 0.997 | 80.5 °C |
| | DWV-B1806 | CTTTTCTAATTCAACTTCACC | | | | |
| ABPV | ABPV-F6548 | TCATACCTGCCGATCAAG | 197 | 2.043 | 0.987 | 81.0 ºC |
| | KIABPV-B6707 | CTGAATAATACTGTGCGTATC | | | | |
| IAPV | IAPV-F6627 | CCATGCCTGGCGATTCAC | 203 | 1.938 | 0.998 | 81.0 °C |
| | KIABPV-B6707 | CTGAATAATACTGTGCGTATC | | | | |
| KBV | KBV-F6639 | CCATACCTGCTGATAACC | 200 | 1.905 | 0.995 | 81.5 °C |
| | KIABPV-B6707 | CTGAATAATACTGTGCGTATC | | | | |
| SBV | SBV-qF3164 | TTGGAACTACGCATTCTCTG | 335 | 2.046 | 0.975 | 80.3 °C |
| | SBV-qB3461 | GCTCTAACCTCGCATCAAC | | | | |
| BQCV | BQCV-qF7893 | AGTGGCGGAGATGTATGC | 294 | 2.194 | 0.983 | 80.5 °C |
| | BQCV-qB8150 | GGAGGTGAAGTGGCTATATC | | | | |
| RNA250 | RNA250-F | TGGTGCCTGGGCGGTAAAG | 227 | 1.962 | 0.999 | 84.0 °C |
| | RNA250-R | TGCGGGGACTCACTGGCTG | | | | |
| RP49 mRNA | RP49-qF | AAGTTCATTCGTCACCAGAG | 205 | 1.902 | 0.999 | 77.6 ºC |
| | RP49-qB | CTTCCAGTTCCTTGACATTATG | | | | |

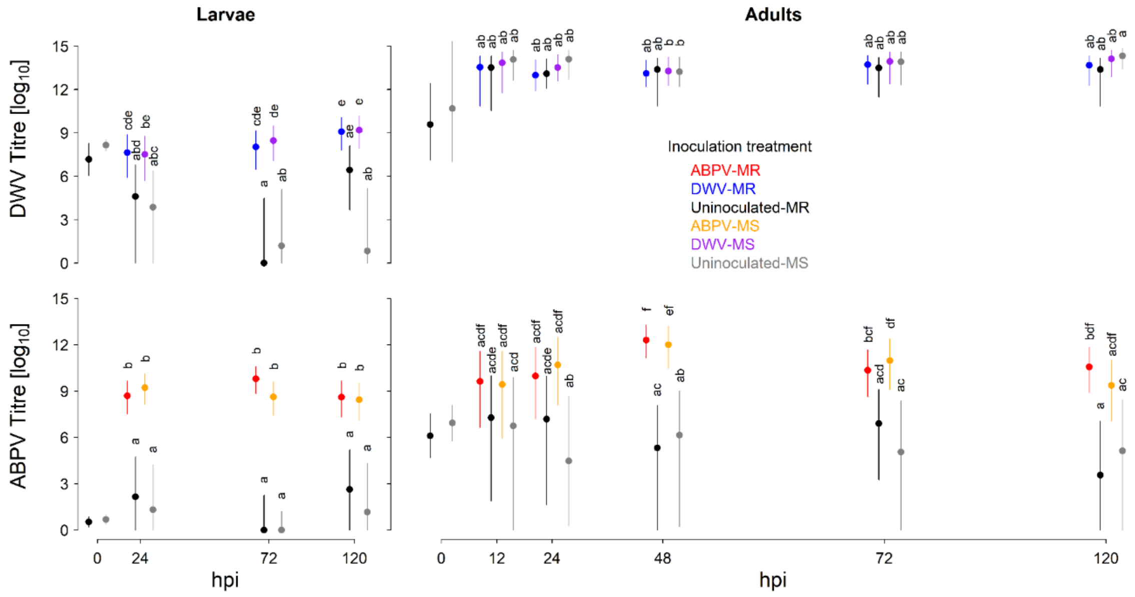


**Figure S1:** Model predictions of titres of deformed wing virus (DWV) and acute bee paralysis virus (ABPV) found in honeybee larvae (left panel) and adult workers (right panel). Mean values are shown with respect to inoculation treatments (DWV, ABPV, uninoculated) and colony history (MR = mite-resistant, MS = mite-susceptible) over the course of the experiment (HPI = hours post inoculation). Different letters indicate significant differences between predicted marginal means with confidence limits from the respective models that still included all explanatory variables and their interactions.

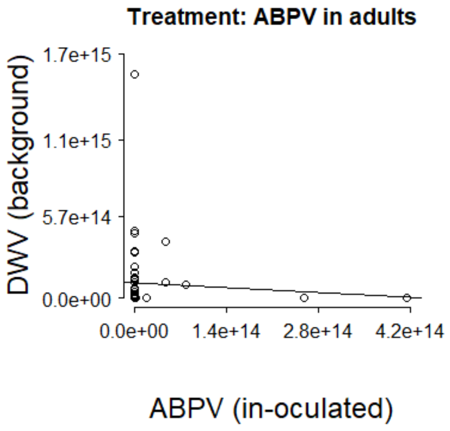


**Figure S2: DWV titres depending on APBV titres**. Line shows linear prediction.

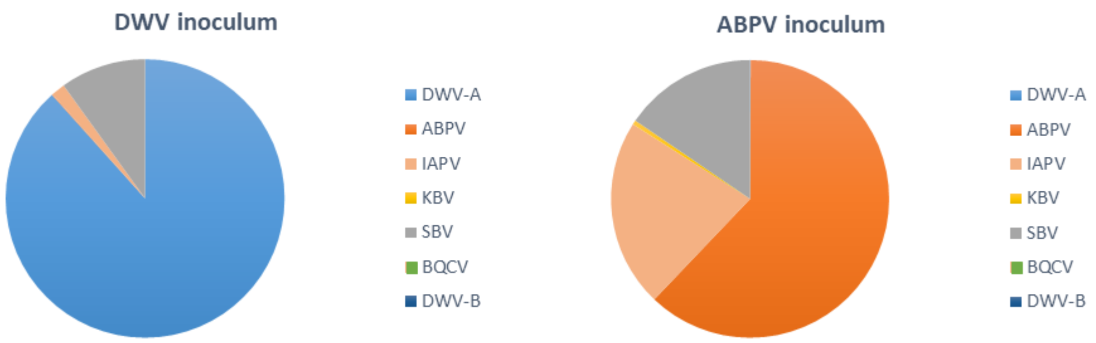


**Figure S3**. The virological composition of the DWV-A and ABPV inocula, based on the most common viruses that can be propagated by injection.

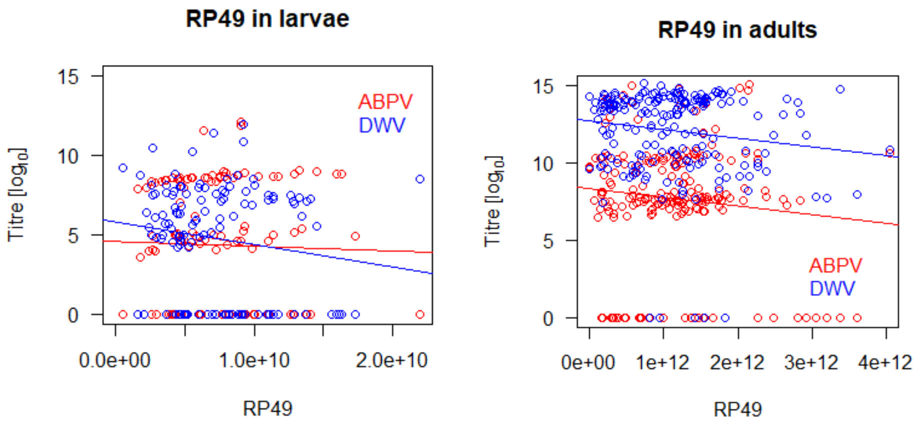


**Figure S4: DWV and ABPV titres depending on RP49 titres.** Lines shows linear prediction from linear regression models of all data for larvae ($DWV^{0.075}$: F=1.94, p=0.16, $R^2$ = 0.01; $ABPV^{0.075}$: F=0.02, p=0.87, $R^2$ = 0.0002) and alive/dead adults ($DWV^{0.125}$: F=3.41, p=0.07, $R^2$ = 0.016; $ABPV^{0.075}$: F=1.36, p=0.24, $R^2$ = 0.006).
